# FDA Risk Assessment of Seafood Contamination after the BP Oil Spill

**DOI:** 10.1289/ehp.1104539

**Published:** 2012-02-01

**Authors:** Robert W. Dickey

**Affiliations:** FDA Gulf Coast Seafood Laboratory, Dauphin Island, Alabama, E-mail: Robert.Dickey@fda.hhs.gov

In response to the BP oil spill of 2010, Rotkin-Ellman et al. (2012) provide a thoughtful assessment of the Food and Drug Administration (FDA) risk criteria to protect vulnerable populations from exposure to polycyclic aromatic hydrocarbons (PAHs) through seafood consumption (FDA 2010a). The FDA and the interagency partners involved in developing the seafood safety risk assessment for the BP oil spill shared the authors’ goal of establishing criteria that would be protective of all affected populations. Although Rotkin-Ellman et al. present a well-intentioned case for determining PAH levels for at-risk populations, their interpretation of several factors involved in the derivation of levels of concern (LOC) differs substantially from those used in the BP oil spill risk assessment.

Human health risk assessments for environmental chemical contaminants are undertaken to develop exposure levels that are believed to be safe or associated with negligible risk. Uncertainty is inherent in any risk assessment process due to interspecies, intraspecies, and/or high-to-low dose extrapolations required for risk estimation. As a result, chemical risk assessments tend to be conservative with intentional bias on the side of safety. Unnecessarily conservative risk criteria, however, can have unintended negative consequences for human health and society. The role of risk management is to balance what is known and unknown about a particular risk with the interests of public health protection and societal values.

Development of the seafood safety risk assessment after the BP oil spill was a collaborative, highly iterative, and multiagency process including departments of health from the five states bordering the Gulf of Mexico. The criteria agreed upon for PAHs with cancer end points provide conservative estimates of contamination levels and consumption rates that, if sustained for a period of 5 years, may result in a upper bound consumer lifetime cancer risk of 1 × 10^–5^. The agreed upon criteria for PAHs with noncancer end points are U.S. Environmental Protection Agency (EPA) reference doses for daily exposure of general and sensitive populations expected to have no significant risk of adverse effect during a lifetime of exposure (U.S. EPA 2000).

A lifetime cancer risk level of 1 × 10^–5^ implies a risk management decision to accept no more than a conservative estimate of one additional cancer case attributable to PAH-contaminated seafood in a population of 100,000 people. This risk level is within the acceptable range of values (i.e., 1 × 10^–4^ to 1 × 10^–6^) observed by public health risk managers (U.S. EPA 1998a, 2000). Bias toward safety is also inherent in the derivation and selection of a benzo[*a*]pyrene cancer potency factor (7.3 mg/kg/day) based on the 95% upper confidence limit of the dose–response curve, rather than a maximum likelihood estimate. The actual cancer risk is believed to be somewhere below this upper confidence limit, and could be as low as zero (U.S. EPA 1994). The U.S. EPA value is more conservative than recent derivations of benzo[*a*]pyrene cancer potency factors, which incorporate adjustments for exposures during infancy (Office of Environmental Health Hazard Assessment 2010).

In regard to specific issues raised about selected underlying assumptions used in the risk assessment process, the FDA respectfully disagrees with the arguments of Rotkin-Ellman et al. (2012). Various assumptions must be made in risk assessments in order to extrapolate data from animal or human studies using models to estimate population risks. Such models are designed to be overly protective to account for uncertainty and variability (e.g., upper 95% confidence bound on cancer risk values, and inter- and intraspecies uncertainty factors on reference doses for noncarcinogens). Within this context, some numerical assumptions are based on average or mean values (e.g., average adult body weight and averaging time), and other assumptions are based on upper percentile values such as annualized food consumption rates.

For purposes of risk assessment, average adult body weight may be viewed as an estimate of average lifetime body weight—and averaging time as average lifespan—of people comprising a population. Because these factors are all condensed into a single number, there is often a range of values that it may be reasonable to use. Risk calculations include these factors in the derivation of contaminant thresholds for lifetime cancer risk and risk of adverse effect during a lifetime of exposure for noncancer end points. The resulting threshold values reflect risk across the average lifespan of a population, including men, women (including pregnant women), and children.

Seafood consumption data are collected for different purposes using a variety of survey instruments. It is important to note that surveys most often reflect short-term intake and do not necessarily address seasonality or otherwise directly capture annualized seafood consumption. The BP risk assessment (FDA 2010a) used National Health and Nutrition Examination Survey (NHANES) 90th percentile consumption data for seafood eaters-only adjusted for consumption frequency (Centers for Disease Control and Prevention 2007). Meal portion and frequency (16.4 seafood meals/month) were converted to annualized daily equivalents.

The selection of 5 years for projected exposure duration following the BP oil spill was considered appropriate and conservative in consideration of the nature of the spilled oil (i.e., light crude), physical conditions (e.g., 29.5°C water temperature), offshore location of the spill (50 miles), and metabolic capacities of seafood species potentially impacted. Exposure duration values selected in previous oil spill assessments range from 2 years (e.g., *New Carissa*, Oregon, 1999) to 10 years (*Exxon Valdez*, Alaska, 1989). Corresponding fishery closures range from weeks to > 6 years for select species that were subjected to prolonged exposures (e.g., farmed salmon, burrowing lobster). In one of the areas most heavily contaminated after the *Exxon Valdez* spill (Windy Bay), benzo(*a*)pyrene equivalents decreased to nondetectable levels by 2.2 years after the contamination event (Bolger et al. 1996). Fisheries closures due to the BP oil spill range from 2 weeks for areas experiencing little to no impact to > 15 months for heavily impacted areas. The reopening of Gulf fisheries based on PAH chemical surveillance results confirmed that selection of a 5-year exposure duration was indeed appropriate and conservative (FDA 2010b; National Oceanic and Atmospheric Administration 2010).

The classification of naphthalene as a noncancer risk in the BP oil spill seafood safety risk assessment was based on current information and concurrence from the U.S. EPA (FDA 2010a). The U.S EPA (1998b) classified naphthalene in “Group C, a possible human carcinogen. This is based on inadequate data of carcinogenicity in humans exposed to naphthalene via the oral or inhalation routes, and the limited evidence of carcinogenicity in animals via the inhalation route.” No oral slope factor or inhalation unit risk estimate were derived for naphthalene by the U.S. EPA because of the weakness of the evidence that naphthalene may be carcinogenic to humans (U.S. EPA 1998b). More recent evaluations have maintained the noncancer risk classification of naphthalene, as evidenced by the 64th meeting of the Food and Agriculture Organization of the United Nations and the World Health Organization (FAO/WHO) Joint Expert Committee on Food Additives (JECFA), which considered but did not include naphthalene among genotoxic and carcinogenic PAHs evaluated (FAO/WHO 2006).

Although well intentioned, the LOC values suggested by Rotkin-Ellman et al. (2012) do not appear to take into account the natural background occurrence of PAHs in foods in many categories. Assessments from the body of scientific literature is perhaps best represented by the deliberations of JECFA (which included experts from the FDA), which found benzo(*a*)pyrene from dietary intake alone to range from 0.16 to 3.3 µg/person/day (Benford et al. 2010; FAO/WHO 1991, 2006). The LOC values proposed by Rotkin-Ellman et al. (2012) would unnecessarily exclude many food groups from consumers, where nutritional benefits far outweighs negligible risk from PAHs.

Public health authorities are responsible for protecting consumers from contaminated commercial and recreational seafood sources, and to that end advisories may be issued to protect consumers. The federal and state interagency risk assessment for seafood safety following the BP oil spill of 2010 was designed and agreed on by all participants to provide conservative criteria that protect the public. The alternative interpretation provided by Rotkin-Ellman et al. (2012) carries a risk of doing more harm than good.
